# Enhancer RNA Transcription Is Essential for a Novel CSF1 Enhancer in Triple-Negative Breast Cancer

**DOI:** 10.3390/cancers14071852

**Published:** 2022-04-06

**Authors:** Michael W. Lewis, Kamila Wisniewska, Caitlin M. King, Shen Li, Alisha Coffey, Michael R. Kelly, Matthew J. Regner, Hector L. Franco

**Affiliations:** 1 The Lineberger Comprehensive Cancer Center, University of North Carolina at Chapel Hill, Chapel Hill, NC 27599, USA; lewismw@email.unc.edu (M.W.L.); kamila@med.unc.edu (K.W.); caitlin_king@med.unc.edu (C.M.K.); shenli@email.unc.edu (S.L.); coffeya@email.unc.edu (A.C.); mkelly95@live.unc.edu (M.R.K.); regnerm@live.unc.edu (M.J.R.); 2 Bioinformatics and Computational Biology Graduate Program, University of North Carolina at Chapel Hill, Chapel Hill, NC 27599, USA; 3 The Department of Genetics, School of Medicine, University of North Carolina at Chapel Hill, Chapel Hill, NC 27599, USA

**Keywords:** breast cancer, ovarian cancer, enhancer, eRNA, CRISPR-Cas9, dCas9-KRAB, Cas13, gene expression

## Abstract

**Simple Summary:**

In cancer, regulatory regions of the genome are hijacked by the tumor cells for the activation of oncogenes that lead to cancer initiation and progression. One of the most salient regulatory elements of the genome are called enhancers, which are characterized by their ability to increase the expression of their target genes. In this study, we identified a novel enhancer that drives the expression of the oncogene CSF1 in triple-negative breast cancer patients, the most aggressive subtype of breast cancer. We demonstrate that this enhancer is specifically active in triple-negative breast cancer patients compared to other breast cancer subtypes and show that its target genes portend a worse clinical outcome in patients. We then use innovative CRISPR-based genome engineering techniques to systematically perturb various features of this enhancer to elucidate its mechanisms of action and determine the consequences on tumor cell growth. Furthermore, we test our model for CSF1 enhancer function in ovarian cancer cells and demonstrate that our findings can apply to other cancer types. These results demonstrate the significant impact of enhancers in cancer biology and highlight their potential as tractable targets for therapeutic intervention.

**Abstract:**

Enhancers are critical regulatory elements in the genome that help orchestrate spatiotemporal patterns of gene expression during development and normal physiology. In cancer, enhancers are often rewired by various genetic and epigenetic mechanisms for the activation of oncogenes that lead to initiation and progression. A key feature of active enhancers is the production of non-coding RNA molecules called enhancer RNAs, whose functions remain unknown but can be used to specify active enhancers de novo. Using a combination of eRNA transcription and chromatin modifications, we have identified a novel enhancer located 30 kb upstream of Colony Stimulating Factor 1 (CSF1). Notably, CSF1 is implicated in the progression of breast cancer, is overexpressed in triple-negative breast cancer (TNBC) cell lines, and its enhancer is primarily active in TNBC patient tumors. Genomic deletion of the enhancer (via CRISPR/Cas9) enabled us to validate this regulatory element as a bona fide enhancer of CSF1 and subsequent cell-based assays revealed profound effects on cancer cell proliferation, colony formation, and migration. Epigenetic silencing of the enhancer via CRISPR-interference assays (dCas9-KRAB) coupled to RNA-sequencing, enabled unbiased identification of additional target genes, such as RSAD2, that are predictive of clinical outcome. Additionally, we repurposed the RNA-guided RNA-targeting CRISPR-Cas13 machinery to specifically degrade the eRNAs transcripts produced at this enhancer to determine the consequences on CSF1 mRNA expression, suggesting a post-transcriptional role for these non-coding transcripts. Finally, we test our eRNA-dependent model of CSF1 enhancer function and demonstrate that our results are extensible to other forms of cancer. Collectively, this work describes a novel enhancer that is active in the TNBC subtype, which is associated with cellular growth, and requires eRNA transcripts for proper enhancer function. These results demonstrate the significant impact of enhancers in cancer biology and highlight their potential as tractable targets for therapeutic intervention.

## 1. Introduction

Breast cancer is the most frequently diagnosed cancer and the leading cause of cancer-related deaths in women worldwide [1,2]. This disease is separated into distinct subtypes defined by the expression of estrogen receptor (ER), progesterone receptor (PR), and/or human epidermal growth factor receptor 2 (HER2) within the tumor [3,4]. Triple-negative breast cancer (TNBC) lacks expression of all three receptors and is distinguished by aggressive growth and poor prognosis [5]. Moreover, there are no approved targeted therapies for TNBC, and chemotherapy remains the standard of care. While TNBC can be sensitive to chemotherapy, approximately 40% of TNBC patients will have a recurrence after initial treatment that is significantly more likely to occur within the first three years and is associated with reduced survival [6,7,8,9]. Therefore, TNBC patients often develop visceral metastases sooner than the other subtypes, and median survival rarely exceeds 12 months after metastasis, highlighting the need to find novel targets for therapeutic interventions [10]. Molecular characterization of TNBC has identified a characteristic and reliable pattern of gene expression, despite a high level of heterogeneity [4,11,12]. However, there is a paucity of known driver mutations (aside from P53 mutations) that has made it difficult to develop effective targeted therapies [13,14]. This suggests that mutations or epigenetic alterations that lie outside of protein-coding regions, possibly within regulatory elements contained in the non-coding regions of the genome, are contributing to the TNBC phenotype.

Enhancers are often found in both inter- and intragenic regions of the genome and are characterized by their ability to drive spatiotemporal patterns of gene expression. Enhancers are commonly defined as regions of DNA that increase the expression of target genes in a distance and orientation-independent manner. Though enhancers are a key component of healthy development and gene regulation, mounting evidence suggests that enhancers can be rewired or hijacked by cancer cells for the activation of genes that promote tumor formation, metastasis, and resistance to therapy [15,16,17]. This is especially true for cancers that have few or no known driver mutations within protein coding genes, such as TNBC. Enhancers are marked by an enrichment of transcription factor binding, open chromatin accessibility, and distinctive histone modifications on flanking nucleosomes [18,19,20]. Histone 3 lysine 4 monomethylation (H3K4me1) is a reliable marker for enhancers, though it does not differentiate between the active or poised state [21,22,23,24]. The addition of Histone 3 lysine 27 acetylation (H3K27ac), however, is associated with active enhancers which have been shown to form chromatin loops that bring the target promoter and enhancer into close spatial proximity for physical interaction [25,26,27,28,29]. This interplay is thought to be essential for enhancer function, though the specific requirements for the formation and purpose of the chromatin loop is not well defined at all loci. These features and combinations thereof have enabled researchers to annotate novel enhancers that drive critical gene expression pathways and has resulted in considerable evidence demonstrating that enhancers are frequently repurposed as part of oncogenesis, particularly through the upregulation of proliferation genes to confer a growth advantage [15,16,17,30,31,32,33,34,35]. Therefore, enhancers represent an area of cancer research that could significantly impact the diagnosis and treatment of TNBC and will likely be extensible to other forms of cancer.

One of the most significant discoveries for elucidating enhancer function has been the transcription of non-coding RNAs from active enhancers (eRNAs) [36,37]. This unexpected revelation altered our understanding of enhancer mechanisms, and though we still have limited knowledge regarding the purpose of eRNA production, key studies have suggested that eRNAs are vital to enhancer function [38,39,40,41,42,43,44,45]. Studies have found that the majority of enhancers can transcribe eRNAs either uni- or bidirectionally, these transcripts average 2–5 kb, and most lack polyadenylation leading to a high turnover rate. Of note, previous reports have demonstrated that eRNA production by RNA polymerase II temporally precedes the activation of target gene promoters [26,36,42,46,47,48]. Whether the act of transcription or the eRNA transcripts themselves are most important for enhancer function has been heavily debated; however, growing evidence suggests that eRNAs may act in cis to support the function of their associated enhancers through a variety of potential roles [40,49,50,51]. The most likely mechanisms of function are through transcription factor binding, chromatin loop stabilization, or serving as a decoy for transcriptional repressors. Regardless of the mechanisms employed, the expression of eRNAs highly correlates with the formation of enhancer-promoter loops and many additional features of active enhancers [26,47,50,52]. Therefore, enhancer transcription represents the most reliable indicator of enhancer activity and can accurately predict novel functional regulatory regions.

In this study, we sought to describe novel enhancers that might contribute to the pathogenicity of TNBC and could improve our understanding of disease progression. We leveraged eRNA transcription and hallmark chromatin modifications measured in breast cancer cell lines to identify an eRNA-producing enhancer that is uniquely active in the TNBC subtype. We then employed an integrated set of CRISPR-based techniques to identify the target genes of this enhancer and couple these results with biological assays to determine its significance in the context of the disease. We use novel Cas13 RNA-guided RNA-degradation approaches to demonstrate that the eRNA transcripts themselves are required for the regulation of the target genes, suggesting a post-transcriptional role in enhancer function. Finally, we extend our findings beyond breast cancer by demonstrating that enhancer transcription and eRNA transcripts are necessary for CSF1 enhancer function in ovarian cancer. Our results propose that this mechanism is extensible to other eRNA-producing enhancers and can be significant to other cancer types.

## 2. Materials and Methods

### 2.1. Cell Culture

All cell lines were obtained from the American Type Culture Collection (ATCC) and were maintained and used for experiments in the Franco lab at UNC Chapel Hill. MDA-MB-436 cells were grown in Dulbecco’s Modified Eagle Medium (DMEM) (Sigma cat. #30-2002) supplemented with 10% fetal bovine serum (FBS) (Sigma) and 1% penicillin/streptomycin (Corning). Media for dCas9-KRAB expressing cell lines and PspCas13b expressing cell lines were supplemented with 1 µg/mL blasticidin (Corning, 30100RB) for maintenance after selection. RfxCas13d expressing cells were maintained in DMEM supplemented with 0.1 µg/mL puromycin (Sigma, P9620) after selection. Cell lines expressing both dCas9-KRAB and multiplexed sgRNAs were grown in DMEM supplemented with 1 µg/mL blasticidin and 0.1 µg/mL puromycin after selection. All other breast cancer subtype cell lines were grown in DMEM supplemented with 10% FBS and 1% penicillin/streptomycin. MCF10A cells were grown in DMEM/F-12 (Life Technologies Inc. cat #11330032) supplemented with 5% horse serum (Life Technologies Inc. cat #16050122), 10 µg/mL insulin, 0.5 µg/mL hydrocortisone, 20 ng/mL recombinant EGF (Lonza cat#: CC-4136), and 1% penicillin/streptomycin. All cells were grown as adherent cultures at 37 °C and with 5% CO_2_. Low passage cell stocks were kept frozen in liquid nitrogen and thawed for use. Before use, cells were authenticated with Short Tandem Repeat profiling through ATCC. All cell lines were tested for mycoplasma.

### 2.2. Global Run-On Sequencing (GRO-Seq) and ChIP-Seq

GRO-seq data and eRNA annotations were downloaded from NCBI’s Gene Expression Omnibus repository under the following accession number GSE96859 (GRO-seq) [16]. Enhancer annotations across all breast cancer subtypes and ChIP-seq data were downloaded from GEO under the following accession GSE85158 [53]. These data were used to rank TNBC-specific enhancers on the basis of H3K27ac enrichment and eRNA expression [16,53]. Candidate TNBC-specific enhancers were then visualized using the UCSC Genome Browser (http://genome.ucsc.edu) and the enrichment of H3K27ac and eRNA production was compared across all breast cancer subtypes. Ovarian cancer H3K27ac ChIP-seq data were downloaded from the SRA database using accession number GSE101408 [54]. Ovarian cancer BRD4 ChIP-seq data were downloaded from the SRA database using accession number GSE77568 [55]. Browser tracks were visualized using the Integrative Genomics Viewer [56,57,58].

### 2.3. RT-PCR of Patient Tumors

De-identified snap-frozen human TNBC tumor specimens were obtained through the UNC Tissue Procurement Core Facility (IRB Protocol 18-3330). RNA was extracted from tissues using the RNeasy Mini Kit (Qiagen, 74106) following the manufacturer’s instructions. RNA quality was verified using the Agilent RNA TapeStation. Genomic DNA was removed with the TURBO DNA-free kit (Invitrogen, AM1907) following the manufacturer’s instructions and 1–2 μg of purified RNA was synthesized into cDNA using M-MLV reverse transcriptase (Promega, M1705) with random primers to facilitate eRNA detection. RT-PCR was performed using 5–80 ng of template cDNA with Taq DNA polymerase (NEB, M0267S) following the manufacturer’s protocols. Amplification was performed with the following program: 1 cycle at 95 °C for 2 min, 30–40 cycles at 95 °C for 15 s and 60 °C or 62 °C for 1 min, then held at 10 °C. Primers for eRNA amplification can be found in Table S1. Variations in RT-PCR template amount, annealing temperature, and cycle number were experimentally determined to be optimal for either robustly produced transcripts or low abundance eRNAs.

### 2.4. Quantitative RT-PCR

RNA extracted from cells was further treated to remove genomic DNA using the TURBO DNA-free kit (Invitrogen, AM1907) following the manufacturer’s instructions. cDNA was synthesized using 1–2 µg of RNA and the M-MLV reverse transcriptase (Promega, M1705) with random primers to allow for eRNA detection. The resulting cDNA was analyzed by qRT-PCR with SYBR Green using the QuantStudio 6 Flex System (Applied Biosystems) and the primers listed in Table S1. mRNA and eRNA expression were normalized to GAPDH and/or ACTB and fold change compared to a negative control was determined using the 2^−ΔΔCT^ (Livak) method [59]. All experiments were conducted at least three times to ensure reproducibility. Results are shown as the mean fold change ± standard error of the mean (S.E.M). Statistical analysis was performed in the R environment (https://www.r-project.org/) using a Student’s two-sample t-test. Statistical significance is indicated by * *p* < 0.05, ** *p* < 0.005, and *** *p* < 0.0005. Primer oligonucleotide sequences can be found in Table S1.

### 2.5. Expression of CSF1 across Breast Cancer or All TCGA Cancer Types

To determine the expression of CSF1 mRNA across a broad patient tumor dataset, we used the METABRIC breast cancer dataset via cBioPortal (https://www.cbioportal.org/) [60,61,62]. In total, 1,904 samples were grouped based on clinical BC subtype and plotted to display CSF1 mRNA expression z-scores relative to all samples (log microarray), showing that claudin-low TNBC tumors had the highest expression of CSF1 mRNA. All statistical tests were two-sided, and statistical significance is indicated by * *p* < 0.05, ** *p* < 0.005, and *** *p* < 0.0005. CSF1 expression across all cancer subtypes was analyzed using The Cancer Genome Atlas Program (TCGA Research Network: https://www.cancer.gov/tcga) dataset to examine 10,071 patient samples [13]. Samples were grouped based on cancer type to observe CSF1 expression in RSEM (RNA-Seq by Expectation Maximization). Groups were arranged based on median value in descending order.

### 2.6. TCGA Assay for Transposase-Accessible Chromatin with High-Throughput Sequencing (ATAC-Seq) Data Analysis

ATAC-seq data for this analysis were downloaded from the TCGA repository Firebrowse (http://firebrowse.org/). TCGA patient barcodes were used to subset down to breast cancer only and then grouped based on PAM50 subtype classification. Bedtools intersect (one bp overlap) was used to identify ATAC peaks overlapping the CSF1 enhancer or transcription start site (TSS) and the mean of the ATAC scores for each region were calculated to represent chromatin accessibility. Statistical analysis was performed with a two-sample Wilcoxon rank sum test.

### 2.7. CSF1 Enhancer Knockout with CRISPR-Cas9

Targeted deletion of the CSF1 enhancer was accomplished using the CRISPR-Cas9 system following published protocols [63,64,65]. Briefly, guide RNA target sites flanking the outer boundaries of the eRNA transcripts were designed using the CRISPOR web tool [66]. Genomic coordinates for the CSF1 enhancer, sgRNA sequences, and genotyping PCR primer sequences can be found in Table S1. Guide RNA oligos were ordered from Integrated DNA Technologies, annealed, and cloned into pSpCas9 (BB)-2A-Puro (PX459) V2.0 (Addgene Plasmid #62988). To ensure deletion of the enhancer region, two sgRNAs (one 5′ and one 3′ of the eRNA peaks) were transfected into MDA-MB-436 or MDA-MB-468 cells using the Fugene 6 transfection reagent (Promega, E2691) following the manufacturer’s protocol. CRISPR-Cas9 positive clones were identified through puromycin selection that began 3 days post-transfection and lasted a total of 7–10 days. To confirm the enhancer deletion, individual positive clones were picked and expanded into separate wells and genotyped via PCR using primers flanking the deletion site (to identify deletion alleles) or primers internal to the enhancer (to identify wild type alleles). Successful CSF1 enhancer deletion resulted in a ~1510–2100 bp deletion and correct knockout cell lines were further analyzed by Sanger DNA sequencing to determine the precise boundaries of the deletion.

### 2.8. Cell Proliferation Assays

Cells were plated in 6-well plates at a density of 25,000 cells/well in DMEM containing 10% FBS and 1% penicillin/streptomycin. Cells were fixed with 10% formaldehyde on days 2, 4, and 6 after seeding and stained with 0.1% crystal violet in 20% methanol. Cells were destained with 10% glacial acetic acid, then destain was transferred to a 96-well plate and read at an absorbance of 595 nm to identify differences in cell proliferation rates across the timepoints. Statistical analysis was performed using a Student’s two sample *t*-test.

### 2.9. Cell Migration Assays

Cells suspended in serum-free DMEM media containing 1% penicillin/streptomycin were plated in a PET cell culture insert (Fisher, 353097) and placed in a Falcon 24 well companion plate (Fisher, 353504) at a density of 150,000 cells/well in a 100 µL volume. The lower chamber beneath the membrane was filled with 500 µL of DMEM media containing 1% P/S and 10% FBS as a chemoattractant. After 48 h the cells were fixed and stained using the Hema 3 kit (Fisher, 122-911). Ten brightfield images were taken of each membrane per replicate and were brightened to increase the contrast between stained cells and membrane background. Image color was inverted using ImageJ (1.53 K) and then uploaded into cell profiler (version 4.2.1) and analyzed with a cell counting pipeline that counted stained cells based on size and signal intensity. Counted migrated cells were averaged per replicate and statistical analysis was performed using a Student’s two sample *t*-test.

### 2.10. Colony Formation Assay

Cells were plated in 6-well plates at a density of 450 cells/well in DMEM containing 10% FBS and 1% penicillin/streptomycin. Cells were grown for ~20 days to allow for colony formation and then fixed with 10% formaldehyde. Colonies were stained with 0.5% crystal violet in 20% methanol and allowed to dry. Images were taken of each well, and colonies were counted using ImageJ with a consistent set of criteria applied to each well. Threshold values of 20 and 170 were applied to each well to highlight colonies and counting was performed using particle analysis set to ignore any colonies smaller than 200 pixels. Statistical analysis was performed using a Student’s two sample *t*-test.

### 2.11. Engineering MDA-MB-436 and OVCAR3 dCas9-KRAB Expressing Cells

Lentivirus containing the Lenti-dCas9-KRAB-blast vector [67] (Addgene #89567) was packaged in HEK-293T cells. HEK-293T cells were seeded in a T75 flask and transfected with the following plasmids: 6.67 µg Lenti-dCas9-KRAB-blast, 5 µg psPAX2 (Addgene #12260), and 3.33 µg PMD2G (Addgene #12259) using Fugene 6 (Promega, E2691) following the manufacturer’s protocol. The lentivirus-containing supernatant was harvested 48- and 72-h post-transfection, and lentivirus was concentrated using Lenti-X Concentrator (Takara, 631231) following the manufacturer’s protocol. Low passage MDA-MB-436 or OVCAR3 cell lines were seeded in 6-well plates at ~50,000 cells/well and independently transduced with harvested lentivirus in DMEM media with 10% FBS and 10 µg/mL polybrene (Millipore, TR1003G). Transduced cells were incubated with lentivirus for 72 h, then placed in DMEM selection media with 5 µg/mL blasticidin for 7 days. Batch-selected dCas9-KRAB cells were verified by Western blot. For Western blot analysis, cells were lysed using the following lysis buffer: 50 mM Tris HCl (pH 8), 0.5 M NaCl, 1% NP-40, 0.5% sodium deoxycholate, 0.1% SDS and 1× protease inhibitor. The primary antibodies used for Western blotting were as follows: Anti-beta Tubulin Loading Control (Abcam, ab6046), Anti-Cas9 Antibody (7A9-3A3) (Santa Cruz Biotechnology, sc-517386). β-tubulin antibody was used at a 1:1500 dilution in 5% BSA in TBST with overnight incubation at 4 °C. The Cas9 antibody was used at a 1:1500 dilution in 5% BSA in TBST with overnight incubation at 4 °C. The secondary antibodies used for Western blotting were as follows: Donkey anti-rabbit IgG, Whole Ab, HRP-conjugated (GE Healthcare, NA934) and Donkey anti-Mouse IgG (H+L), and HRP-conjugated (Thermo Fisher Scientific, PA1-28748). Secondary antibodies were used at a 1:5000 dilution in 5% BSA in TBST. Original uncropped Western blots shown in Figure S3.

### 2.12. CRISPRi sgRNA Design and Vector Cloning

sgRNAs targeting the CSF1 enhancer or transcription start site were designed using the CRISPOR web tool [66]. Several sgRNAs targeting unique regions of the enhancer or TSS were designed to be transfected together (Table S1). A previously published non-targeting scrambled sgRNA was used as a negative control [68]. The sgRNA cloning vector, pX-sgRNA-eGFP-MI, is a modified version of pSpCas9 (BB)-2A-Puro (pX459) v2.0 (Addgene #62988) [69]. Cas9 was removed from pX459 and replaced with eGFP to enable visualization of sgRNA expression. To improve sgRNA stability and optimize for assembly with dCas9, the sgRNA stem-loop was extended and modified with an A-U base pair flip [70]. sgRNA vector cloning was performed following the protocol from Feng Zheng’s group [69]. Briefly, sgRNA oligonucleotides ordered from Integrated DNA Technologies (IDT) were duplexed with the following reaction: 10 µM sgRNA forward oligo, 10 µM sgRNA reverse oligo, 10 U T4 polynucleotide kinase (NEB, M0201L), and 1× T4 ligation buffer under the following conditions: 37 °C for 30 min, 95 °C for 5 min, then ramp down to 25 °C at 5 °C/min. Duplexed sgRNAs were diluted 1:100 and 2 µL of this dilution was used in a ligation reaction with 100 ng pX-sgRNA-eGFP-MI linearized with BbsI-HF (NEB, R3539S). The ligation product was transformed into Subcloning Efficiency DH5alpha Competent Cells (Invitrogen, 18265017) following the manufacturer’s protocol. Each completed sgRNA vector was verified by Sanger sequencing using a human U6 promoter sequencing primer. This primer and the sgRNA oligonucleotide sequences can be found in Supplementary Materials Table S1.

### 2.13. CRISPR Interference (CRISPRi) Targeted Perturbation

Cell lines that stably express sgRNAs were created using a lentiviral multiplexed vector that was a gift from Anna Chiarella in the Hathaway lab at UNC Chapel Hill. This vector allows for stable expression of several sgRNAs at once under the control of the human U6 promoter. Lentiviral vectors were cloned to either contain a single non-targeting scrambled sgRNA or a combination of four sgRNAs all targeting the CSF1 enhancer. Lentivirus was created and collected following the same protocol used for engineering dCas9-KRAB expressing cells described above. Low passage MDA-MB-436-dCas9-KRAB cell lines were seeded in 6-well plates at ~50,000 cells/well and independently transduced with harvested lentivirus in DMEM media with 10% FBS and 10 µg/mL polybrene (Millipore, TR1003G). Transduced cells were incubated with lentivirus for 72 h, then placed in DMEM selection media with 1 µg/mL puromycin for 15 days. Batch-selected MDA-MB-436-dCas9-KRAB-sgRNA cells were screened for multiplexed sgRNA vector insertion via genotyping PCR (Supplementary Materials Table S1).

OVCAR3 dCas9-KRAB interference transient transfection experiments were performed following published protocols with modifications [71]. dCas9-KRAB expressing cells were seeded 1 day prior to transfection in 6-well plates at 200,000 cells/well in antibiotic-free DMEM. Cells were transfected with 1.5 µg sgRNA vector per well using Fugene 6 at a 2:1 Fugene:DNA ratio (Promega, E2691) following the manufacturer’s protocol. A single scrambled sgRNA vector was used as a negative control. A combination of 2–4 targeting sgRNA plasmids were co-transfected (in equal ratio) per well and visualized for GFP expression. MCF7 dCas9-KRAB transient transfection experiments were performed as described above, except dCas9-KRAB expression vector was pooled with the sgRNAs during transfection as the MCF7 cells did not stably express dCas9-KRAB. RNA was extracted from cells 48–72 h post-transfection by washing with 1× PBS and processing with the Zymo Quick-RNA Miniprep Kit (Zymo, R1055) and on-column DNaseI treatment. RNA was further treated for genomic DNA removal using the TURBO DNA-free kit (Invitrogen, AM1907). All experiments were performed three or more times to ensure reproducibility and statistical fidelity.

### 2.14. RNA-Sequencing and Analysis

RNA was collected from MDA-MB-436-dCas9-KRAB cells subjected to CRISPRi targeting as described above. RNA-seq library preparation and sequencing was performed by the Novogene Company (novogene.com). Libraries were prepared using the Illumina TruSeq mRNA-seq kit following the manufacturer’s instructions and were sequenced by paired-end 150 bp sequencing on the Illumina Novaseq 6000 platform. RNA-sequencing was aligned to the hg38 genome using STAR 2.7.6a and quantified with Salmon 1.4.0. All samples were evaluated to ensure sequencing passed acceptable quality thresholds for analysis by utilizing the Picard 2.22.4 CollectRnaSeqMetrics function and FastQC. Differential expression analysis was performed using the DESeq2 package in R. Differentially expressed genes were defined as any gene with an adjusted *p*-value < 0.1 and a mean expression >20 (DESeq2 mean of normalized counts for all samples). RNA-seq data were uploaded to GEO under the following accession number GSE185318.

### 2.15. Kaplan Meier Survival Analyses in Breast Cancer and Ovarian Cancer

Breast cancer Kaplan–Meier curves were created in the R environment (packages rms and survival) using the METABRIC RNA expression and survival datasets (overall survival) subset to only contain breast cancer patients [60]. Patients were separated into two groups of high or low gene expression using a median-centered cutoff. Hazard ratios (HR) were calculated using the Cox proportional hazards model and P values were determined with a log-rank test. All statistical tests were two-sided and the significance level was set to *p* < 0.05. Ovarian cancer KM curves were generated using the Kaplan–Meier Plotter web tool (https://kmplot.com/analysis/) [72]. CSF1 (209716_at) was selected as the Affy id/gene symbol and only JetSet best probe set were used to examine 1435 patients. All other options were set at default.

### 2.16. Engineering PspCas13b Stable Expression Cell Lines

The pC0046-EF1a-PspCas13b-NES-HIV (Addgene #103862) was modified to enable Cas13b endonuclease activity within the nucleus for specific eRNA transcript targeting. This was accomplished by removing the HIV nuclear export signal (NES) and replacing it with the SV40 nuclear localization sequence (NLS). A nucleoplasmin NLS was also inserted immediately upstream of the Cas13b coding sequence. Finally, a T2A self-cleaving peptide and Blasticidin resistance cassette was inserted for the selection of positive clones and the 3× HA tags were conserved. This resulted in the Lenti-PspCas13b-NLS-HA-Blast vector for eRNA degradation. Stable Cas13b expression cells were created for MDA-MB-436 and OVCAR3 cells following the same methods as described in the engineering dCas9-KRAB cells section. The primary antibody for Western blot was anti-HA tag (Sigma, SAB4300603).

### 2.17. PspCas13b eRNA Knockdown Transfections

Guide RNA sequences were designed using an online Cas13 design tool (https://cas13design.nygenome.org/) and were cloned into pC0043-PspCas13b crRNA backbone (Addgene #103854) following the methods previously described (Table S1) [73]. Cells stably expressing Cas13b were seeded in 6-well plates at 350,000 cells/well (MDA-MB-436) or 200,000 cells/well (OVCAR3) in antibiotic-free media 1 day prior to transfection. Cells were transfected with 1.5 µg sgRNA vector per well using Fugene 6 (Promega, E2691) following the manufacturer’s protocol. A scrambled sgRNA vector was used as a negative control. A combination of 2–4 targeting sgRNA plasmids were co-transfected (in equal ratio) per well. RNA was extracted from cells 24 h post-transfection by washing with 1× PBS and processing with the Zymo Quick-RNA Miniprep Kit (Zymo, R1055) and on-column DNaseI treatment. RNA was further treated for genomic DNA removal using the TURBO DNA-free kit (Invitrogen, AM1907). All experiments were performed three or more times to ensure reproducibility and statistical fidelity.

### 2.18. Engineering RfxCas13d Stable Expression Cell Lines

The pLentiRNACRISPR_005-hU6-DR_BsmBI-EFS-RfxCas13d-NLS-2A-Puro-WPRE vector (Addgene #138147) was cloned to contain either a non-targeting scrambled sgRNA (negative control) or an sgRNA targeting the CSF1 eRNA (positive strand) following the previously described methods (Supplementary Materials Table S1). Guide RNA sequences were designed using an online Cas13 design tool (https://cas13design.nygenome.org/) [73]. Stable expression cell lines were created using these vectors and following the same methods as described in the engineering dCas9-KRAB cells section. Transduced cells were batch selected using DMEM with 0.5% puromycin and positive cell lines were confirmed using genotyping PCR with primers specific to RfxCas13d (Table S1).

### 2.19. RNA Interference (RNAi)

Cells were seeded in 6-well plates at 350,000 cells per well in antibiotic-free DMEM media. After 24 h, cells were transfected with 40 nM of siRNA (IDT DsiRNA) using 3 µL RNAiMAX (Invitrogen, 13778075) following the manufacturer’s protocol. After 48 h, wells were washed with 1× PBS, and RNA was extracted using the Zymo Quick-RNA Miniprep Kit (Zymo, R1055) with on-column DNaseI treatment. RNA was further treated for genomic DNA removal using the TURBO DNA-free kit (Invitrogen, AM1907). Experiments were performed three or more times to ensure reproducibility and statistical fidelity. siRNA sequences can be found in Supplementary Materials Table S1.

### 2.20. Apoptosis Assay of MDA-MB-436 CSF1 Enhancer Knockout Cells

Apoptotic cells were identified by Annexin V-Alexa Fluor and Propidium iodide (PI) staining using the Alexa Fluor^®^ 488 Annexin V/Dead Cell Apoptosis Kit (ThermoFisher Scientific cat #V13241) following the manufacturer’s protocol with minor modification. Briefly, MDA-MB-436 CSF1 enhancer knockout and wildtype cells were collected and washed with 1× PBS. Cells were counted and aliquoted to contain 100,000 cells in a 100 µL volume. Aliquots were stained with 5 µL Alexa Fluor^®^ 488 annexin V and 1 µL 100 µg/mL PI. Cells were incubated at room temperature for 15 min before adding 400 µL 1× annexin-binding buffer and mixing gently. Cells were kept on ice until they could be analyzed by flow cytometry measuring the fluorescence emission at 530 nm and >575 nm.

## 3. Results

### 3.1. Enhancer RNA Production Highlights a Novel Enhancer of CSF1 in Triple-Negative Breast Cancer (TNBC)

**Figure 1:**
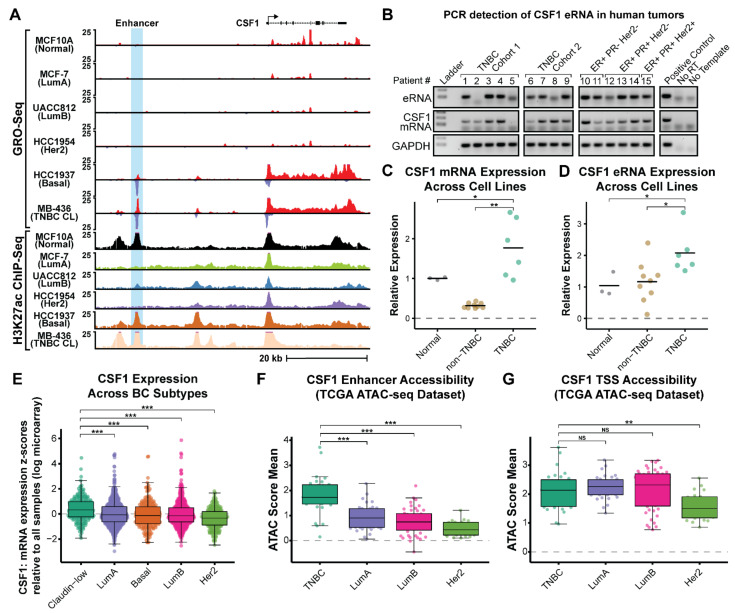
eRNA transcription and its associated epigenomic modifications identify a novel eRNA producing enhancer upstream of *CSF1* in triple-negative breast cancer. (**A**). GRO-seq (red peaks indicate transcription in the sense strand and blue peaks indicate transcription in the anti-sense strand) and H3K27ac ChIP-seq browser tracks showing eRNA production and H3K27ac enrichment at the CSF1 enhancer (highlighted in blue) across multiple breast cancer cell lines. (**B**). RT-PCR of human tumors collected from TNBC and non-TNBC patients to confirm the expression and detection of eRNA (top) and mRNA (middle) transcripts. GAPDH (bottom) expression is shown as a loading control. The positive control (MDA-MB-436 cDNA), reverse transcriptase negative control, and no template control are shown on the right. C and D. qRT-PCR showing changes in CSF1 mRNA expression (**C**) and CSF1 eRNA expression (**D**) across cell lines grouped by normal mammary epithelial cells (gray), non-TNBC cancer cell lines (yellow), and TNBC specific cell lines (green). Each point represents the fold change (relative to the normal group) for an individual replicate and the bar shows the mean value for each group. Significance determined by a two-sided *t*-test comparing each KO line to WT (* *p* < 0.05, ** *p* < 0.01). (**E**). Boxplot comparison of CSF1 mRNA expression across the METABRIC cohort of breast cancer patients, grouped by subtype. Each point represents an individual patient sample. Significance determined by a two-sided *t*-test comparing each KO line to WT (*** *p* < 0.001). (**F**). Boxplot of mean ATAC-seq scores demonstrating increased chromatin accessibility of the novel CSF1 enhancer in basal/claudin-low (TNBC) subtype breast cancer patients as compared to other subtypes. Each point represents the mean ATAC-seq score for a single library across the 5 bins spanning the CSF1 enhancer region. Asterisks indicate significant difference when comparing each group to TNBC (Wilcoxon rank-sum test, *** *p* < 0.001). (**G**). Boxplot of mean ATAC-seq scores overlapping the CSF1 transcription start site (TSS) demonstrating consistent accessibility across breast cancer subtypes within the TCGA cohort. ** *p* < 0.01, The designation of NS represents statistically not significant.

Enhancer activity and eRNA production are highly cell-type-specific [45]. We and others have shown that actively transcribed enhancers are more likely to associate with enhancer-related chromatin modifications (such as H3K4me1 and H3K27ac), loop to target gene promoters, and correlate with target gene activation [45]. Thus, enhancer transcription is a good predictor of active enhancers and can be used in the absence of other genomic information to predict active enhancers de novo [16]. To this end, we mined existing Global Run-On coupled sequencing (GRO-seq) and Chromatin Immunoprecipitation with sequencing (ChIP-seq) data across 13 different breast cancer cells lines to identify TNBC-specific eRNA transcription [16]. We reasoned that using signatures of enhancer transcription would allow us to identify active enhancers that are breast cancer subtype-specific and potentially drive the expression of cancer-relevant genes. One of the most highly expressed eRNAs in TNBC cells is located ~30 kb upstream of the Colony Stimulating Factor 1 gene (CSF1) and shows strong enrichment of H3K27ac, supporting its designation as an active enhancer region (Figure 1A). While H3K27ac signal is present in other breast cancer subtypes at this site, we found that eRNA transcription is specific to TNBC cells and correlates with increased expression of its nearest neighboring gene, CSF1 (Figure 1A). This is in agreement with recent literature suggesting that eRNA production is a robust indicator of enhancer activity and is likely more reliable than the presence of H3K27ac alone [47,74,75]. The putative target gene for this enhancer, CSF1, encodes a growth factor that regulates proliferation and differentiation of the monocytes-macrophage lineage through binding its receptor, a cell-surface tyrosine kinase called CSF1R [76,77,78]. In breast cancer, CSF1 stimulates tumor-associated macrophages (TAMs) that contribute to tumor initiation and progression by promoting angiogenesis, extracellular matrix breakdown, and tumor cell motility [79,80,81,82,83]. Moreover, CSF1 (and CSF1R) expression has been shown to correlate with progression and clinical outcome in breast cancer, making this enhancer an interesting candidate for further study [84,85,86,87,88].

We next sought to confirm our GRO-seq analysis using qRT-PCR across the breast cancer cell lines. We chose representative cells from each subtype and combined them into groups based on their classification as normal immortalized epithelial cell lines (MCF10A), non-TNBC cell lines (MCF7, HCC1954, and UACC812), or TNBC-specific cell lines (MDA-MB-436 and HCC1937). Our results confirmed that we could detect significantly more CSF1 mRNA and eRNA in the TNBC cell lines when compared to normal or non-TNBC (Figure 1C,D). To determine if these transcripts could also be detected in human tumors, we procured nine samples collected from TNBC patients and six from non-TNBC patients for analysis of CSF1 expression. We performed reverse transcriptase PCR (RT-PCR) with primers specific to either eRNA or mRNA transcripts, demonstrating that both could be detected in human tumors (Figure 1B). However, variations in RNA quality, time of storage for samples, and the quality of each tumor specimen obtained made it challenging to quantitatively measure the differences in eRNA expression levels across this small sample size of human tumors. Instead, we leveraged much larger datasets to examine the quantitative changes in the expression of CSF1 mRNA across BC patients. We found that CSF1 is most highly expressed in TNBC Claudin-low patients and is significantly higher than the next highest subtype, Luminal A (*p*-value = 9.436 × 10^−6^, Welch two sample *t*-test) (Figure 1E) [60]. To determine if the activity of the enhancer might be contributing to the increased CSF1 expression in TNBC tumors, we analyzed The Cancer Genome Atlas (TCGA) ATAC-seq data to compare the chromatin accessibility landscape of this enhancer across 133 breast cancer samples [89]. Patients were separated into groups based on BC subtype (PAM50 classification), and accessibility scores were calculated based on the mean ATAC-seq score for the five bins overlapping the enhancer region. We found that the CSF1 enhancer is significantly more accessible in TNBC patients as compared to the HER2, LumA, and LumB groups (*p*-values of 2.25 × 10^−9^, 3.02 × 10^−7^, and 8.59 × 10^−10^, respectively) (Figure 1F). This demonstrates that enhancer accessibility, which is required for enhancer function, is greater in the TNBC group. Interestingly, the CSF1 mRNA transcription start site (TSS) showed similar chromatin accessibility in the TNBC group compared to LumA and LumB groups and while exhibiting more accessibility compared to the HER2 group (*p*-values of 2.28 × 10^−3^, 0.23, and 0.46, respectively). This suggests that the CSF1 mRNA promoter is viable in all subtypes and the enhancer may be responsible for amplifying the expression in TNBC patients (Figure 1G). Taken together, these results suggest that a novel regulatory element ~30 kb upstream of the CSF1 gene is more highly transcribed in triple-negative breast cancer patients and is associated with increased expression of CSF1 mRNA.

### 3.2. Deletion of the CSF1 Enhancer Reduces CSF1 mRNA and Leads to Growth Defects in TNBC Cells

**Figure 2:**
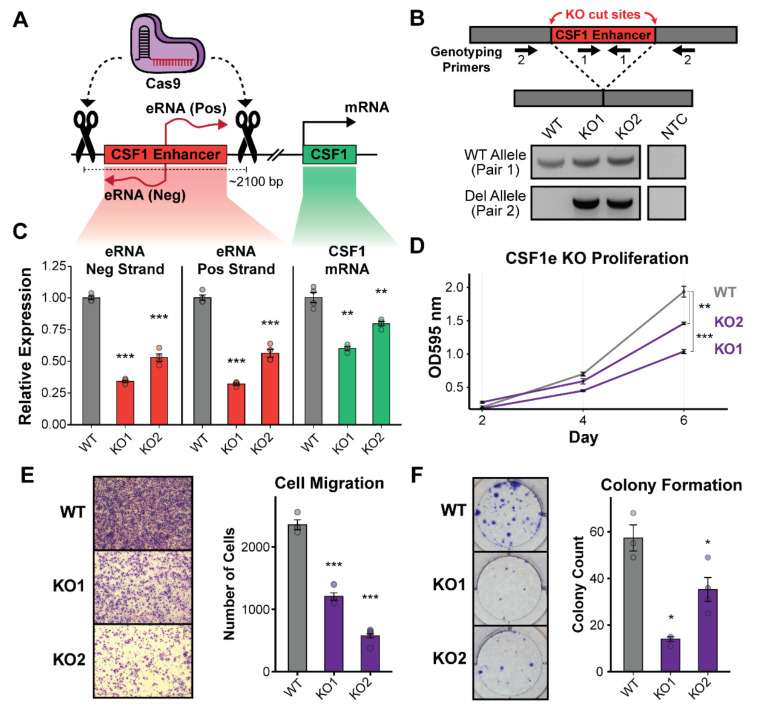
Knockout of the CSF1 enhancer confirms cis-regulatory activity and leads to reduced cell proliferation, migration, and colony formation. (**A**). Diagram showing the use of Cas9 and flanking sgRNAs to target and cut regions flanking the CSF1 enhancer, resulting in a ~2100 bp deletion. (**B**). Genotyping was performed by PCR amplification both within the WT allele and across the deletion site to create PCR products representative of genomic deletion (top). Genotyping PCR of CSF1e-KO clones confirms multiple heterozygous knockouts (bottom). (**C**). qRT-PCR analysis showing that CSF1 enhancer RNAs (both positive and negative strand) and the CSF1 mRNA are significantly reduced in heterozygous KO clones. Bars show mean fold change (relative to WT cell line) in CSF1 eRNAs (negative strand left, positive strand middle) or mRNA (right); points represent fold change values for individual replicates. Error bars are standard error of the mean. *n* = 4. Statistical significance determined by a two-sided *t*-test comparing each KO line to WT (** *p* < 0.01, *** *p* < 0.001) (**D**). Proliferation assay shows a significant reduction in cell growth following deletion of the CSF1 enhancer. Each point shows the mean absorbance reading for each cell line across all four replicates. Error bars standard error of the mean. Significance determined by a two-sided *t*-test comparing each KO line to WT (** *p* < 0.01, *** *p* < 0.001). (**E**). Migration assays of KO clones demonstrate a reduced potential for migration when the CSF1 enhancer has been deleted. Brightfield images (left) show the presence of stained cells on migration columns. Bars represent the mean number of cells counted (relative to WT cell line) across all replicates per cell line (right). Individual points show number of cells counted per field of view captured. Error bars are standard error of the mean. *n* = 4 with 10 fields counted per replicate (*** *p* < 0.001). (**F**). Colony formation assays show a reduction in ability to form colonies from a single cell following deletion of the CSF1 enhancer. Representative well images show a decrease in colony formation for CSF1e-KO cell lines (left). Bar plots show results of mean colony counts for each cell line across all four replicates (right). Individual points are shown for each replicate. Significance determined by a two-sided *t*-test comparing each KO line to WT (* *p* < 0.05).

To determine if this eRNA-producing enhancer is a bona fide enhancer of CSF1, we employed a CRISPR-Cas9 approach to knockout the genomic region containing the enhancer in the TNBC claudin-low cell line, MDA-MB-436. This cell line was chosen because CSF1 expression is highest in this cell line as determined by GRO-seq (Figure 1A); it is also highest in claudin-low TNBC patients (Figure 1E). A combination of four single guide RNAs (sgRNAs) were designed to deliver Cas9 to the 5′ and 3′ flanking regions of the enhancer (two sgRNAs per flank), resulting in a ~2100 bp deletion that removed the entirety of the transcribed enhancer (Figure 2A). The resulting CSF1 enhancer knockout (CSF1e-KO) clones were individually genotyped for the presence of a deletion allele and/or WT allele (Figure 2B). No clones were found to be homozygous for deletion of the CSF1 enhancer, which we suspect may have been lethal to the cells; however, several heterozygous deletion clones were identified, and two (CSF1e-KO1 and CSF1e-KO2) were selected for further investigation (Figure 2B). Quantitative reverse transcriptase PCR (qRT-PCR) was used to measure the changes in CSF1 eRNA and mRNA expression in wild type vs. CSF1e-KO cells. We found that the deletion of at least one CSF1 enhancer allele led to a significant reduction in both positive and negative strand eRNA transcription (Figure 2C). This also resulted in a significant decrease in CSF1 mRNA, suggesting that this region is acting to amplify expression of the CSF1 gene. To confirm these results in an independent cell line, we generated CSF1 enhancer knockouts in the MDA-MB-468 TNBC basal cell line with the same sgRNAs. Using qRT-PCR, we confirmed the results observed in the MDA-MB-436 cell line by showing a comparable decrease in CSF1 mRNA expression when the enhancer was deleted (Supplementary Materials Figure S1A). To examine if the enhancer is providing growth or migration advantages to TNBC cells, the CSF1e-KO cell lines were assayed for changes in biological phenotypes. Measuring the proliferation potential of CSF1e-KO cells compared to WT over a 6-day time course showed a significant reduction in growth associated with the deletion of the enhancer (Figure 2D). We next explored if enhancer loss could have an impact on cancer cell motility. Transwell migration assays using only fetal bovine serum as a chemoattractant displayed a significant decrease in cell movement when comparing CSF1e-KO to WT (Figure 2E). Finally, we investigated whether the CSF1 enhancer could be providing cells with a growth advantage by promoting survival and expansion of single cells into larger colonies. To address this, we employed a colony formation or clonogenic assay and found that enhancer deletion correlated with a significant reduction in colony-forming potential compared to WT TNBC cells (Figure 2F). To determine if these observations could be the result of cell death induced by enhancer knockout, we performed an apoptosis assay using propidium iodide and Annexin V-Alexa Fluor staining combined with flow cytometry. This experiment revealed a small increase in early apoptotic cells (Annexin V+/PI−) and dead cells (Annexin V+/PI+) but does not show enough change to explain the phenotype changes observed in previous assays (Supplementary Materials Figure S1B–D). Taken together, these results demonstrate that this regulatory element is a true enhancer of the CSF1 gene and that CSF1 overexpression could be contributing to the aggressive growth characteristics observed in TNBC cells.

### 3.3. Expression and Accessibility of the CSF1 Enhancer Is Critical for Its Activity

**Figure 3:**
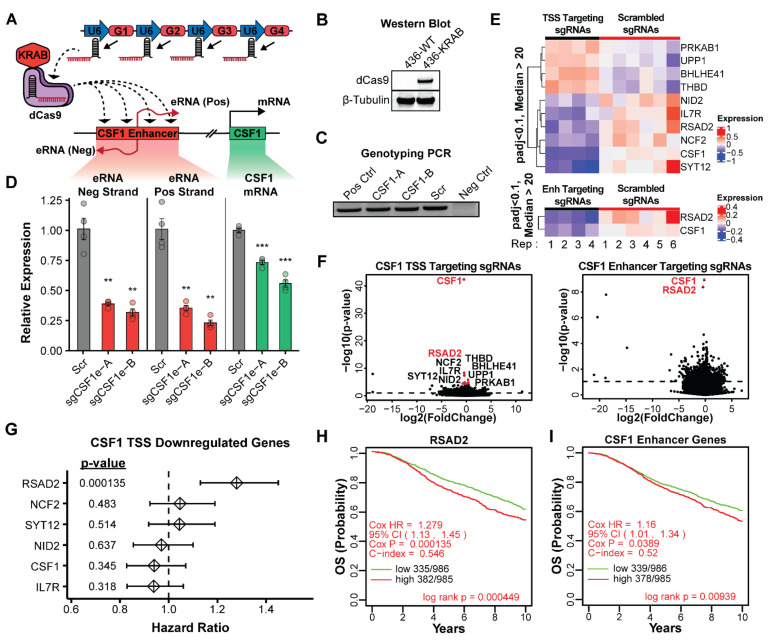
Epigenetic silencing of the CSF1 enhancer reduces CSF1 mRNA expression and identifies target genes significant to patient outcome. (**A**). Diagram showing the approach for CRISPRi epigenetic silencing of the CSF1 enhancer. A construct containing four guide RNAs targeting distinct areas of the CSF1 enhancer was transduced into dCas9-KRAB expressing MDA-MB-436 cells, facilitating targeting of the enhancer for epigenetic repression. (**B**). Western blot showing expression of dCas9-KRAB protein in cell lines used for this work. β-tubulin is shown below as a loading control. (**C**). Genotyping PCRs demonstrating insertion of the guide RNA expression vector within TNBC cell lines. (**D**). qRT-PCR showing changes in CSF1 eRNA or mRNA expression resulting from dCas9-KRAB targeting of the CSF1 enhancer. CSF1-A and CSF1-B are independent cell lines (biological replicates) expressing dCas9-KRAB and the same sgRNA expression vector. Bars represent mean fold change (relative to scrambled sgRNA expressing cell line) and points show individual fold change values for each replicate. Significance determined by a two-sided *t*-test comparing each KO line to WT (** *p* < 0.01, *** *p* < 0.001). *n* = 4. (**E**). RNA-seq heatmap displaying the changes in gene expression resulting from CRISPRi perturbation of the CSF1 transcription start site (top) or enhancer (bottom). CSF1 TSS or enhancer targeting replicates shown on the left, *n* = 4. Negative control scrambled replicates shown on the right, *n* = 6. (**F**). Volcano plots showing differential gene expression (*p*-value versus fold change difference) results by CRISPRi perturbation of the CSF1 transcription start site (left) or enhancer (right). Genes shown in red are significantly downregulated. (**G**). Forest plot showing hazard ratios for the genes downregulated in the CSF1 TSS targeting samples. (**H**). Kaplan–Meier plot showing patient overall survival probability separated by high or low expression (median centered) of RSAD2 using the METABRIC patient cohort. (**I**) Kaplan–Meier plot showing patient overall survival probability separated by high or low expression of CSF1 and RSAD2.

Enhancer transcription is a hallmark of activity. To test whether transcription of the CSF1 enhancer is critical for its function in TNBC cells, we employed a CRISPR interference (CRISPRi) approach to epigenetically repress the enhancer region while leaving the genomic DNA intact. This technique relies on a catalytically deactivated version of Cas9 (dCas9) fused to the KRAB repressor domain that can be targeted to specific genomic loci using a complementary guide RNA (sgRNA) [90]. Once bound to a specific genomic locus, the KRAB domain mediates the recruitment of TRIM28 (tripartite motif-containing protein 28), serving as a scaffold for the binding of other repressive factors [91]. Heterochromatin formation and transcriptional repression are facilitated through the ensuing binding of SETDB1 (SET domain bifurcated 1) [92], the NuRD complex [93,94], and HP1 (heterochromatin protein 1) [95,96] that coordinate to remove histone acetylation, establish H3K9me3, and promote DNA methylation resulting in robust epigenetic silencing of the local chromatin environment [97,98]. For this experiment, we created two independent cell lines that stably express dCas9-KRAB and four guide RNAs (sgRNAs) that specifically target dCas9-KRAB to the CSF1 enhancer (sgCSF1e-A and sgCSF1e-B) (Figure 3A). As a negative control, we created an additional cell line that stably expresses dCas9-KRAB and a non-targeting scrambled sequence guide RNA. As quality control, the expression of dCas9-KRAB was confirmed via Western blot (Figure 3B), and the expression of the appropriate guide RNAs was confirmed via genotyping PCR (Figure 3C). Genotyping primers were designed to amplify a region of the multiplexed sgRNA vector immediately 5′ to the repetitive sgRNA insertion sites. Quantitative RT-PCR was used to assess changes in eRNA and mRNA expression between scrambled sgRNA cells and CSF1e CRISPRi cells. We found that epigenetic perturbation of the CSF1 enhancer led to a significant decrease in both eRNA transcripts (positive and negative strand), which was accompanied by a reproducible decrease in CSF1 mRNA transcript (Figure 3D). This result indicates that the transcription and accessibility of the enhancer is required for the upregulation of the CSF1 mRNA, demonstrating that epigenetic disruption of this loci is sufficient to inhibit CSF1 transcription. To confirm that this enhancer activity is specific to the TNBC subtype, CRISPRi perturbation of the CSF1 enhancer was also performed on MCF7 (Luminal A) cells using identical sgRNAs. We found no significant change in either CSF1 mRNA or eRNA expression when the enhancer was targeted with dCas9-KRAB in MCF7 cells, suggesting that the enhancer is not active in these cells (Supplementary Materials Figure S2A,B).

To empirically determine all of the potential target genes of the CSF1 enhancer, CRISPRi perturbation was combined with RNA-seq to examine genome-wide expression changes after CSF1 enhancer silencing. As a comparison to the enhancer targeting experiments, we also designed sgRNAs to specifically target the CSF1 mRNA at the transcription start site (TSS). This enabled us to highlight downstream changes associated with a reduction in CSF1 mRNA expression versus the enhancer RNA activity. We found that targeting the TSS of CSF1 led to the downregulation of six genes (CSF1, RSAD2, NID2, IL7R, NCF2, and SYT12) and the upregulation of four genes (PRKAB1, UPP1, BHLHE41, and THBD) using a *p*-value-adjusted cutoff of <0.1 and a base mean expression >20 (DESeq2 mean of normalized counts for all samples) (Figure 3E,F). Epigenetic repression of the CSF1 enhancer resulted in the statistically significant downregulation of two genes, CSF1 and RSAD2 (Figure 3E,F). The presence of CSF1 downregulation in both conditions confirms our previous findings that this enhancer is driving the expression of CSF1. The reduced expression of RSAD2 in both TSS and enhancer-targeting samples suggests a relationship with CSF1 gene expression that had not been previously reported. These results suggest that the enhancer is likely upregulating CSF1 expression and that CSF1 may be part of a more complex network of gene expression.

### 3.4. Activity of the CSF1 Enhancer Is Correlated with Reduced Patient Survival

The evidence collected thus far suggests that the CSF1 enhancer is specific to triple-negative breast cancer, may be conferring growth advantages to tumor cells, and a reduction in CSF1 leads to changes in the expression of several other genes. This led us to speculate that the CSF1 enhancer could be impacting patient outcomes. To address this, we first focused on genes that were downregulated upon CRISPRi inhibition of the CSF1 TSS and calculated hazard ratios (HRs) using the Cox proportional hazards model for each gene using the METABRIC breast cancer dataset (Figure 3G). We found that several genes had a HR of >1 (RSAD2, NCF2, and SYT12), indicating a negative impact on patient outcome. RSAD2 was notable for having the largest HR (1.279, *p*-value 1.35 × 10^−4^) and being affected when either the CSF1 TSS or CSF1 enhancer were inhibited. Interestingly, CSF1 alone was not associated with a significant negative outcome in our analysis of this dataset. Focusing on RSAD2, we stratified patients into either high or low expression groups and generated a Kaplan-Meier survival curve to evaluate the impact on overall survival probability (Figure 3H). This showed a significant reduction in patient survival, using a log-rank test, when RSAD2 is upregulated. The Kaplan–Meier survival curve generated using the CSF1 enhancer knockdown associated genes (CSF1 and RSAD2) also shows a reduced patient survival that is statistically significant (Figure 3I). These findings, combined with our biological assays, suggest that activation of the CSF1 enhancer leads to increased expression of CSF1 and additional downstream genes that may be contributing to the aggressive nature of TNBC tumors and negatively impacting patient survival.

### 3.5. Enhancer RNA Transcripts Are Necessary for CSF1 Upregulation in a Post-Transcriptional Mechanism

**Figure 4:**
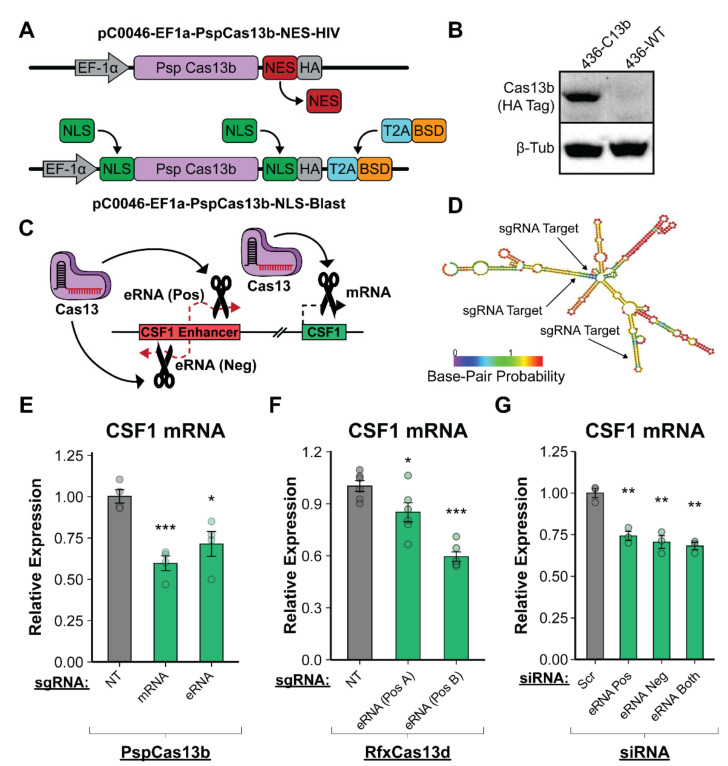
Cas13-targeted disruption of the CSF1 eRNA transcript causes reduction in CSF1 mRNA expression. (**A**). Vector map displaying modifications to pC0046-EF1a-PspCas13b-NES-HIV vector to allow for eRNA targeting within the nucleus. (**B**). Western blot showing expression of the HA tagged PspCas13b protein in MDA-MB-436 cells. (**C**). Diagram showing approach for Cas13b-mediated RNA degradation. Guide RNAs were pooled and transiently transfected to enable targeting of the either the CSF1 eRNAs or the CSF1 mRNA. (**D**). Predicted structure of the positive strand eRNA demonstrating potential secondary structures. Sites selected for sgRNA targeting labeled. (**E**). qRT-PCR displaying the relative fold change in CSF1 mRNA expression when targeting either the CSF1 mRNA or CSF1 eRNAs with PspCas13b. Each bar represents the mean fold change (relative to a non-targeting guide RNA) and each point shows the individual fold change per replicate. Error bars show standard error of the mean. Significance determined by a two-sided *t*-test comparing each KO line to WT (**F**). qRT-PCR displaying the relative fold change in CSF1 mRNA expression when targeting the CSF1 eRNA (positive strand) with RfxCas13d in two independent cell lines (biological replicates). Each bar represents the mean fold change (relative to a non-targeting guide RNA) and each point shows the individual fold change per replicate. Error bars show standard error of the mean. Significance determined by a two-sided *t*-test comparing each KO line to WT (**G**). qRT-PCR showing effects of siRNA-mediated eRNA knockdown on CSF1 mRNA expression. Each bar represents the mean fold change (relative to a scrambled siRNA) and each point shows the individual fold change per replicate. Error bars show standard error of the mean. (* *p* < 0.05, ** *p* < 0.01, *** *p* < 0.001).

With a clinical significance established for this novel enhancer, we next sought to understand how eRNA transcription contributed to the function of the enhancer. There are several models for enhancer function that place varying levels of importance on the eRNA transcripts produced [45]. Some suggest that the act of transcription is sufficient for enhancer function with no direct role for the eRNA transcript itself [99,100]. Others indicate that the transcripts can perform a post-transcriptional role that is necessary for target gene upregulation [38,39,40,42,43,48,49,50,51,101,102,103,104,105,106,107,108,109,110,111,112,113,114]. Further complicating the matter, these studies demonstrate that enhancers can use distinct mechanisms of function at different loci. Of note, while CRISPRi epigenetic repression of the enhancer led to a downregulation of CSF1 mRNA, this does not clarify whether the loss of eRNA production or the disrupted enhancer–promoter interaction caused by KRAB are responsible for this change. It is possible that either of these changes alone or both together are necessary to observe this result. Therefore, separating the opportunity for enhancer–promoter physical interaction and the act of transcription from a post-transcriptional role for eRNAs requires techniques that allow for direct perturbation of transcripts without altering enhancer transcription or the underlying DNA. To address this, we modified and employed a recently described approach using Cas13, an RNA-guided CRISPR enzyme that allows for targeted cleavage of RNA transcripts with a high degree of accuracy and efficiency [115,116,117,118,119]. Cas13 functions similarly to DNA targeting CRISPR enzymes such as Cas9 but instead binds and cleaves RNA transcripts. To facilitate the targeted degradation of eRNA transcripts, we leveraged the pC0046-EF1a-PspCas13b-NES-HIV (Addgene #103862) vector containing the *Prevotella* Sp. (Psp) Cas13b enzyme in a lentiviral vector. We modified this plasmid to enable targeting within the nucleus by removing the nuclear export signal, inserting multiple nuclear localization sequences, and introducing a blasticidin resistance gene for clonal selection (Figure 4A). MDA-MB-436 TNBC cells were transduced with this modified PspCas13b vector to create a stable expression cell line that was confirmed through Western blot using an antibody that recognizes Cas13b via the HA tag (Figure 4B). Target sites for sgRNAs were carefully selected using both the Cas13 Guide Design Resource (https://cas13design.nygenome.org) and RNAfold (http://rna.tbi.univie.ac.at/cgi-bin/RNAWebSuite/RNAfold.cgi) to predict regions of the eRNA transcripts that are likely to form secondary structures that can prevent an sgRNA from recognizing its target sequence (Figure 4D). To mitigate the effects of secondary RNA structures, multiple sgRNAs were designed and pooled per target transcript. The transfection of sgRNAs followed by qRT-PCR to examine CSF1 mRNA expression shows that targeting the mRNA directly with Cas13b has the most robust knockdown effect (Figure 4E). This outcome demonstrates that Cas13b is capable of cleaving the CSF1 mRNA for targeted knockdown. Next, we targeted the CSF1 enhancers RNAs (both positive and negative strand) using Cas13b and found that this was sufficient to cause a significant decrease in CSF1 mRNA levels (Figure 4E). This suggests that the CSF1 eRNA transcripts are required post-transcriptionally for mRNA upregulation, as this technique degrades targeted transcripts but does not impact their transcription.

To further bolster our eRNA knockdown studies, we employed a more recently described version of Cas13 (RfxCas13d or CasRx) that is derived from *Ruminococcus flavefaciens*, is smaller than Cas13b, and has been shown to function effectively within the nucleus [120]. To determine if RfxCas13d could reproduce our CSF1 eRNA knockdown results, we created multiple MDA-MB-436 TNBC cell lines that stably express RfxCas13d and a single guide RNA targeting the eRNA positive strand transcript. Using qRT-PCR in two independent cell lines, we found that stably targeting and disrupting the eRNA transcript led to a significant reduction in CSF1 mRNA when compared to a non-targeting scrambled sgRNA, confirming our results using Cas13b (Figure 4F). As an orthogonal approach to the Cas13 experiments, we used siRNA-mediated RNA-interference assays designed to target and degrade either the positive or negative strand eRNAs. Like Cas13b-targeted degradation, this allows for a specific perturbation of the eRNA transcripts apart from the act of transcription. Using qRT-PCR to assess changes in CSF1 eRNA and mRNA levels, we found that the siRNA-mediated degradation of either eRNA transcript led to a decrease in CSF1 mRNA (Figure 4G). This finding confirms the Cas13 results and indicates that the eRNA transcripts are crucial for enhancer function.

### 3.6. The CSF1 Enhancer Is Active in Ovarian Cancer and eRNA Transcription Is Essential for Function

**Figure 5:**
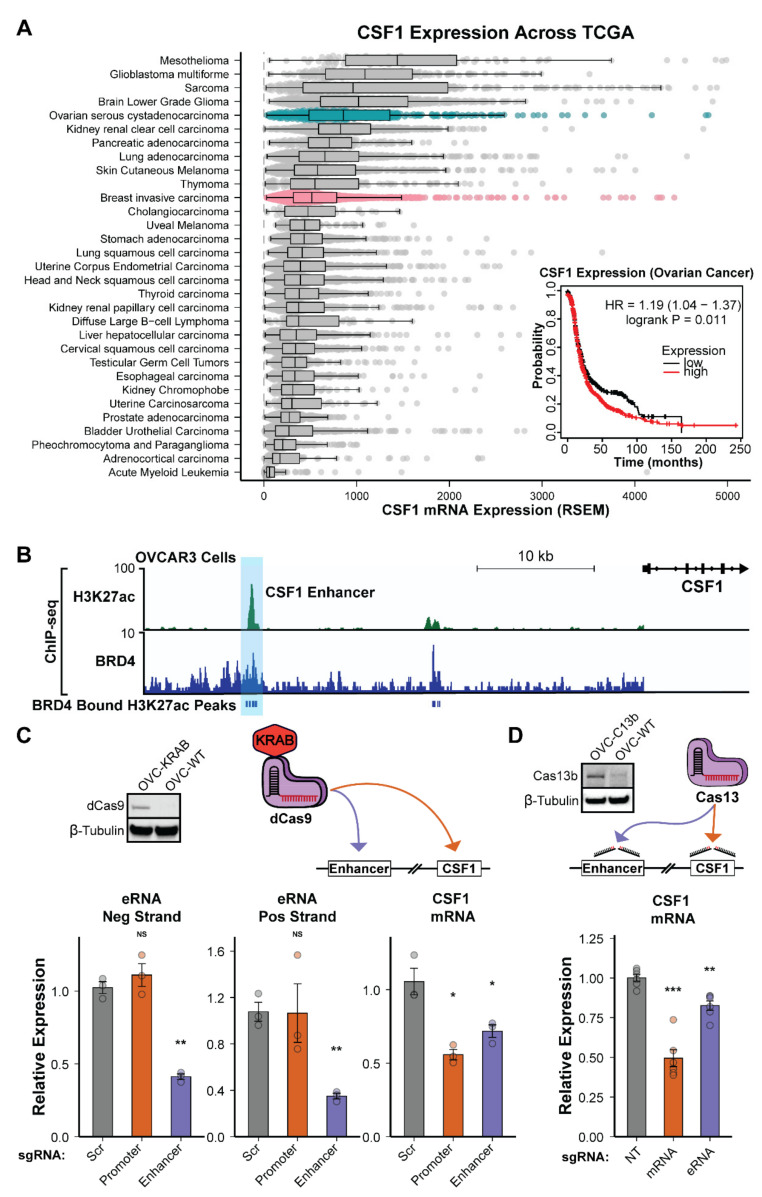
The CSF1 enhancer is active in ovarian cancer patients and requires eRNA transcripts for its activity. (**A**). A Boxplot showing the expression of CSF1 (RSEM) across all cancer types of the TCGA dataset. Cancer types are arranged by median in descending order. Inset: Kaplan–Meier plot showing patient overall survival probability separated by high or low expression of CSF1 in ovarian cancer. (**B**). Browser tracks displaying H3K27ac (green) and BRD4 (blue) ChIP-seq overlapping the CSF1 enhancer and TSS in OVCAR3 High-grade serous ovarian cancer cells. The CSF1 enhancer is highlighted in light blue and BRD4-bound H3K27ac peaks are indicated below. (**C**). Top left: Western blot showing expression of dCas9-KRAB protein in OVCAR3 cells. β-tubulin is shown below as a loading control. Top right: Diagram showing the targeting of dCas9-KRAB to either the enhancer (purple) or the promoter (orange). Bottom: qRT-PCR displaying the relative fold change in CSF1 eRNA (positive and negative strand transcripts) or mRNA expression when targeting the CSF1 enhancer or promoter with dCas9-KRAB. Each bar represents the mean fold change (relative to a scrambled guide RNA), and each point shows the individual fold change per replicate. Error bars show standard error of the mean. Significance determined by a two-sided *t*-test comparing each promoter or enhancer sample to scrambled control (* *p* < 0.05, ** *p* < 0.01), NS represents statistically not significant. (**D**). Top left: Western blot showing expression of the HA tagged PspCas13b protein in OVCAR3 cells. Top right: Diagram showing the targeting of Cas13b to either the enhancer RNA (purple) or the mRNA (orange). Bottom: qRT-PCR displaying the relative fold change in CSF1 mRNA expression when targeting the CSF1 eRNA or mRNA with Cas13b. Each bar represents the mean fold change (relative to a scrambled guide RNA), and each point shows the individual fold change per replicate. Error bars show standard error of the mean. Significance determined by a two-sided *t*-test comparing each promoter or enhancer sample to scrambled control (** *p* < 0.01, *** *p* < 0.001).

To further test the mechanisms of CSF1 enhancer function and determine if our observations about this enhancer are extensible to other cancer types, we surveyed the TCGA Pan-Cancer Atlas dataset to identify other types of cancer in which CSF1 is highly expressed. Interestingly, we found that ovarian cancer was among the highest in CSF1 mRNA expression (RSEM) and even surpassed breast cancer (Figure 5A). In addition, we found that increased CSF1 expression was correlated with a worse outcome in ovarian cancer patients, as determined by a Kaplan–Meier survival curve (Figure 5A). To ascertain if the enhancer was active in ovarian cancer cells and may be responsible for this increased expression, we downloaded publicly available H3K27ac and BRD4 ChIP-seq datasets and looked for the enrichment of these marks at the CSF1 enhancer region (Figure 5B) [54,55]. In the absence of publicly available GRO-seq data, we instead relied on a combination of H3K27ac and BRD4 ChIP-seq to indicate activity of the enhancer. Bromodomain-containing protein 4 (BRD4) is known to be enriched on active enhancers, and combined with H3K27ac, provides a robust method to identify active enhancers [121,122,123]. As seen in Figure 5B, the CSF1 enhancer is highly active in the high-grade serous ovarian cancer cell line OVCAR3. Therefore, to test our model in ovarian cancer cells, we created dCas9-KRAB expressing OVCAR3 cell lines and repeated the CRISPRi experiments using the same sgRNAs to epigenetically repress the CSF1 enhancer. We found that inhibition of the enhancer led to a decrease in CSF1 mRNA and both eRNA transcripts (positive and negative strand), matching the results observed in TNBC cell line experiments (Figure 5C). In this experiment, we also targeted the CSF1 TSS with sgRNAs designed to repress transcription of the mRNA without inhibiting the enhancer. Interestingly, we found that directly targeting the TSS led to the most robust knockdown in mRNA levels but did not affect the expression of either eRNA transcript (Figure 5C). Next, we created Cas13b expressing OVCAR3 cell lines to test the role of the eRNAs directly. As is observed in the TNBC eRNA perturbation experiment, there was a significant decrease in CSF1 mRNA expression upon the degradation of the eRNA transcript (Figure 5D). Taken together, we demonstrate that eRNA transcripts are required for the regulation of CSF1 gene expression, and we posit that this observation could be generalizable to other eRNA-target gene relationships. This work implicates a role for the CSF1 enhancer in the progression of triple-negative breast cancer and potentially other cancer types, highlighting the eRNA transcripts and associated downstream genes as potential targets for the therapeutic intervention.

## 4. Discussion

Research into the networks of genes altered by enhancers in cancer has also exposed the multitude of genomic lesions that can transform these regions. While germline variants are often found to increase the risk of tumor development, they are typically not sufficient to trigger cancer formation [124,125]. Accumulated somatic mutations on top of these germline variants often have more profound effects, such as conferring growth advantages that drive tumor formation [126,127]. Additional mechanisms to activate oncogenes include chromosomal translocations that place them under the control of previously unassociated enhancers, large deletions or rearrangements that connect them with upregulating elements, amplifications that can convert an individual enhancer into a super-enhancer, and more discreet mutations that constitutively activate regulatory regions [33,128,129]. This emphasizes the need for expanded research into non-coding elements in the genome to fully understand the mechanisms of gene regulation and the consequences in cancer. Identification of pathogenic enhancer activity can provide new targets for study and intervention through the elements themselves or the collection of genes they regulate.

In this study, we leveraged eRNA expression to identify a novel enhancer of CSF1 that was active specifically in the TNBC subtype of breast cancer. Patient-derived datasets confirmed that this enhancer is most active in TNBC individuals, and we were able to detect CSF1 eRNA and mRNA transcripts in patient tumor samples. This supports our findings in cell lines and makes them extensible to breast cancer patients. We used Cas9 knockout cell lines to demonstrate that this region is a true enhancer for the CSF1 gene. Surprisingly, heterozygous deletion of the enhancer was sufficient to cause profound defects in cell proliferation, migration, and colony formation, highlighting the biological importance of this enhancer. Since deletion of the enhancer could potentially have unintended genomic consequences, we leveraged CRISPR interference (dCas9-KRAB) assays that allowed us to focus on the necessity for transcription of the enhancer without altering the underlying DNA. Epigenetic silencing of the enhancer caused a reduction in CSF1 eRNA and mRNA expression, indicating that transcription is vital for enhancer function. We also used this approach as an opportunity to identify other targets of the enhancer using CRISPRi-coupled to RNA-seq. We confirmed CSF1 as target gene of the enhancer and identified several genes that appear to be impacted by the CSF1 enhancer, notably RSAD2. While high expression of CSF1 alone is not associated with poor patient outcomes in the datasets we analyzed, previous work indicates that CSF1 is involved with the formation of tumor associated macrophages (TAMs) that are significant for cancer development and progression [79,80,81,82,83,130]. Interestingly, increased expression of RSAD2 is associated with worse survival for breast cancer patients, and to our knowledge, the relationship between CSF1 and RSAD2 has not been shown before. We found that RSAD2 expression decreases when we repress either the CSF1 enhancer or the CSF1 transcription start site, suggesting that RSAD2 is not a target gene of the enhancer but instead depends on the expression of CSF1. This discovery highlights both genes as interesting potential targets for therapeutic intervention and warrants further investigation that is outside the scope of this work.

We next focused our attention on the significance of the eRNA transcripts at the CSF1 enhancer by employing multiple versions of a CRISPR-based RNA-guided RNA-knockdown approach using newly discovered Cas13 effector proteins [131,132]. This technique allows us to test the functions of the eRNA transcripts themselves without affecting the enhancer itself. We used both a modified version of PspCas13b and unmodified RfxCas13d to target and degrade eRNA transcripts originating from the enhancer, which led to a significant reduction in CSF1 mRNA expression. This indicates that the eRNAs are required post-transcriptionally for the function of the enhancer, and these results were confirmed using RNA interference techniques (siRNAs) to achieve similar results. Moreover, we validated this mechanism in ovarian cancer cell lines. Thus, we posit that the CSF1 enhancer RNAs serve a required function in the enhancer mechanism, likely through the binding of transcription factors, stabilizing the enhancer–promoter loop interactions, or via sequestering repressive factors that could inhibit target gene upregulation. While this work does not define the specific function of these transcripts, it demonstrates the significant impact that these non-coding RNAs can have in an aggressive form of cancer. This also confirms that eRNA production is a valuable indicator of enhancer activity and can be leveraged to identify novel enhancers, even in circumstances where the enhancer has been rewired in cancer cells. This work supports other studies suggesting eRNAs serve a functional part of the enhancer mechanism that should be explored further [38,39,42,43,45,48,50,101,102,103,104,105,106,107,108,109]. We know that eRNAs are produced with similar kinetics to their target genes and have a high turnover rate due to degradation by the exosome [36,42,133,134,135,136]. We have also learned that eRNA transcripts can perform several roles for target gene upregulation, which can vary from enhancer to enhancer. This necessitates the investigation of each enhancer/eRNA transcript to confirm its specific utility at a given loci. Interestingly, enhancer RNAs have already been suggested as targets for cancer therapeutic intervention and are likely to play an important role in cancer diagnosis and treatment in the future [137]. This work bolsters these ideas and encourages further investigation into eRNAs and enhancers in the context of cancer, which we believe will provide future avenues for therapeutic intervention.

Though the study of these eRNA transcripts is vital to advance cancer research, their investigation also presents certain challenges that must be overcome to gain meaningful insight. One obstacle is selecting a target site for either Cas13-based guide RNAs or small interfering RNAs (siRNAs). These sites need to be free of secondary structures to facilitate proper targeting and degradation, which typically requires the use of complex structure-prediction software and manually testing several gRNAs [115,116,131]. If using qRT-PCR to examine changes in eRNA levels after targeted degradation, it is also important to consider the location of primers. Transcripts that have been cut at a single site may still be detected as a complete transcript if the PCR primers lie within one segment of the cut RNA. This creates additional considerations for Cas13, which does not yet have a well-established cut site, but instead is thought to cleave at uracil-rich regions near the sgRNA targeting location [116]. This can make primer placement more difficult but may not be a major concern as the enzyme is likely to cut in multiple locations, increasing the likelihood of a break between forward and reverse primers. Another potential issue that should be addressed when targeting eRNA transcripts for knockdown is the kinetics of eRNA transcription and eRNA degradation. We know that most eRNAs are produced at a similar rate to their target genes, but the rate of eRNA degradation when using Cas13 or RNAi is not clear. eRNAs are also predominantly non-polyadenylated and therefore have a very short half-life. Using qRT-PCR to detect changes in eRNA levels after targeted knockdown is inherently difficult as the transcripts are being constantly produced, and the pool that exists in the nucleus is short-lived. While some studies avoid this issue by investigating eRNAs that are polyadenylated or relatively stable by other means, this is likely to be a concern for a majority of eRNA studies moving forward. The establishment of a target gene relationship and using detection of target gene mRNA expression as a readout is one option, but there may be other approaches that could be employed in the future. One potential alternative is the use of small molecule inhibitors, such as flavopiridol to inhibit the transcription of RNA polymerase II, which would prevent continued production of enhancer RNAs and allow for the measurement of the existing pool of eRNAs [138]. However, this still leaves a constantly diminishing pool of transcripts to work with and could have other impacts on the cells not associated with eRNA knockdown. Another potential approach is the inhibition of the exosome, which has been used to create a buildup of enhancer RNAs and allows for a better detection of knockdown efficiency [134,135,136,139,140,141]. This may also lead to non-specific results but could be employed for the sole purpose of estimating eRNA knockdown efficiency for a given technique. Regardless of these caveats, the investigation of enhancer RNAs has proven to be complicated, yet paramount to many fields of research. The work presented here demonstrates the significance of eRNA transcripts and their importance specifically to triple-negative breast cancer and ovarian cancer, which we anticipate will be extensible to other forms of cancer. Understanding how these transcripts enable target gene upregulation and how they can be exploited to detect and/or treat cancer is important for the fields of cancer biology and gene regulation alike.

## 5. Conclusions

Cancer is a challenging disease to study, due in part to the wide variety of mutations and rearrangements that can give rise to pathogenesis. One hallmark of breast cancer is the inappropriate expression of genes, typically driven by regulatory networks that are often rewired through various molecular aberrations. This is best demonstrated by the association between cancer and the unusually high number of mutations within transcription factors. It has also become apparent that cancer cells rely heavily on increased transcription, often referred to as transcriptional addiction, that is necessary to drive pathogenic expression pathways required for continual growth [142,143]. As we continue to learn more about the significant impact of enhancers regulating large networks of genes, we can better understand the potential for deleterious effects when enhancers are rewired. Investigation into how the disruption of large-scale gene regulation can impact disease, specifically cancer, has shown that minor defects in regulatory regions can have profound effects downstream [15]. Developing a thorough understanding of changes in enhancer function that drive pathogenesis of specific cancers will prove critical to developing effective treatments.

## Data Availability

GRO-seq data and eRNA annotations were downloaded from NCBI’s Gene Expression Omnibus repository under the following accession number: GSE96859 (GRO-seq) [16]. Enhancer annotations across all breast cancer subtypes and ChIP-seq data were downloaded from GEO under the following accession GSE85158 [53]. RNA-seq data generated in this study were uploaded to GEO under the following accession GSE185318.

## Supplementary Materials

The following are available online at https://www.mdpi.com/article/10.3390/cancers14071852/s1, Figure S1: CSF1 enhancer knockout in MDA-MB-468 cells and apoptosis assay of MDA-MB-436 CSF1e-KO cells (related to Figure 2). (**A**) qRT-PCR displaying the relative fold change in CSF1 mRNA expression when comparing MB468 CSF1 enhancer KO lines to WT. Each bar represents the mean fold change (relative to a scrambled guide RNA) and each point shows the individual fold change per replicate. Error bars show standard error of the mean. Significance determined by a two-sided t-test comparing each promoter or enhancer sample to scrambled control (*** *p* < 0.001). (**B**–**D**) FACS sorting results of wildtype MDA MB-436 cells (**B**) compared to enhancer knockout cells (**C** and **D**) stained with propidium iodide and Annexin V-Alexa Fluor. The dot plots quantify the ratios of live (quadrant 4, Q4) vs. apoptotic (quadrant 3, Q3) vs. dead (quadrant 2, Q2) cells. Figure S2: CSF1 mRNA expression after dCas9-KRAB perturbation of the enhancer in MCF7 cells (related to Figure 3). (**A**) Diagram showing the targeting of dCas9-KRAB to the enhancer (purple) of CSF1. (**B**) qRT-PCR displaying the relative fold change in CSF1 mRNA and eRNA expression when targeting the CSF1 enhancer with dCas9-KRAB in MCF7 cells. Each bar represents the mean fold change (relative to a scrambled guide RNA) and each point shows the individual fold change per replicate. Error bars show standard error of the mean. Significance determined by a two-sided t-test comparing each promoter or enhancer sample to scrambled control (The designation of NS represents statistically not significant). Figure S3: Original uncropped Western blots shown in main text (related to Figure 3, Figure 4 and Figure 5). (**A**) Uncropped Western blot and densitometry analysis associated with Figure 3B. (**B**) Uncropped Western blot and densitometry analysis associated with Figure 5C. (**C**) Uncropped Western blot and densitometry analysis associated with Figure 4B (MDA-MB-436 Cas13b) and Figure 5D (OVCAR3 Cas13b). Table S1: Oligonucleotides, Primers, and sgRNAs used in this study.
