# Supplementary material for: Enhancer RNA Transcription Is Essential for a Novel CSF1 Enhancer in Triple-Negative Breast Cancer
Source: Cancers (Basel). 2022 Apr 6;14(7):1852. doi: 10.3390/cancers14071852 (PMC8997997; doi:10.3390/cancers14071852)
Supplement: Supplementary file 1 [file cancers-14-01852-s001.zip › cancers-1515825-supplementary/Figures S1-S3.pdf]

cancers

MDPI

Supplementary Materials

Enhancer RNA Transcription is Essential for a Novel CSF1 Enhancer in Triple-Negative Breast Cancer

Michael W. Lewis [1], Kamila Wisniewska [1], Caitlin M. King [1], Shen Li [1], Alisha Coffey [1], Michael R. Kelly [1,2], Matthew J. Regner [1,2] and Hector L. Franco [1,2,3,*]

1 The Lineberger Comprehensive Cancer Center, University of North Carolina at Chapel Hill, Chapel Hill, NC 27599, USA; lewismw@email.unc.edu (M.W.L.); kamila@med.unc.edu (K.W.); caitlin_king@med.unc.edu (C.M.K.); shenli@email.unc.edu (S.L.); coffeya@email.unc.edu (A.C.); mkelly95@live.unc.edu (M.R.K.); regnerm@live.unc.edu (M.J.R.)
2 Bioinformatics and Computational Biology Graduate Program, University of North Carolina at Chapel Hill, Chapel Hill, NC 27599, USA
3 The Department of Genetics, School of Medicine, University of North Carolina at Chapel Hill, Chapel Hill, NC 27599, USA
* Correspondence: hfranco@med.unc.edu

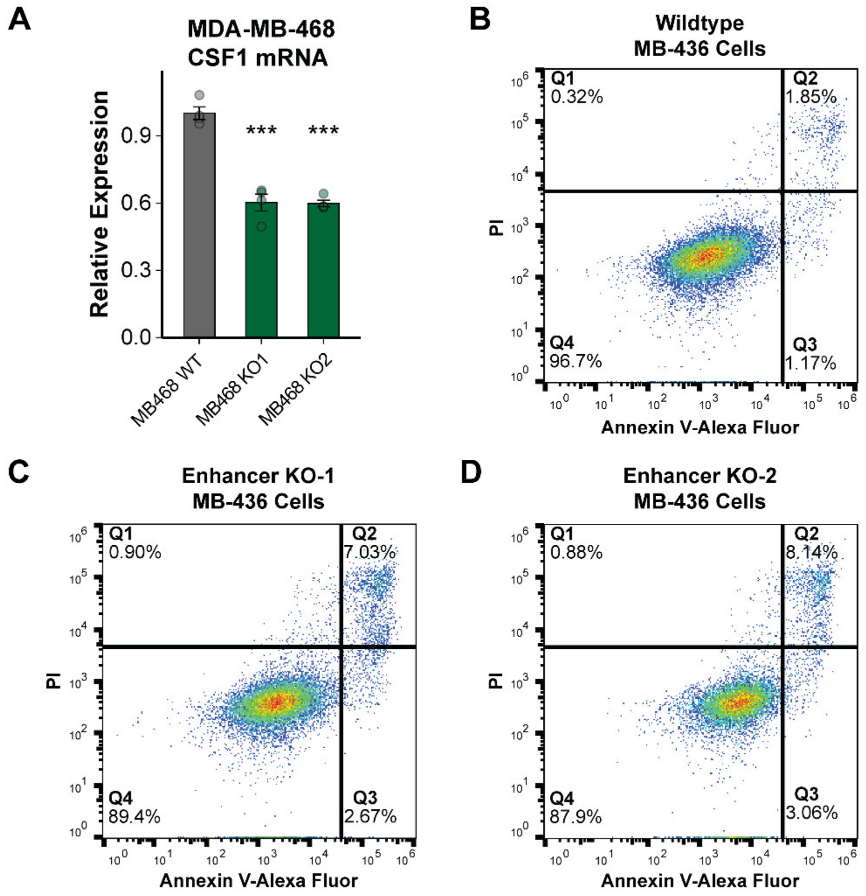

Figure S1. CSF1 enhancer knockout in MDA-MB-468 cells and apoptosis assay of MDA-MB-436 CSF1e-KO cells (related to Figure 2). (**A**) qRT-PCR displaying the relative fold change in CSF1 mRNA expression when comparing MB468 CSF1 enhancer KO lines to WT. Each bar represents the mean fold change (relative to a scrambled guide RNA) and each point shows the individual fold change per replicate. Error bars show standard error of the mean. Significance determined by a two-sided t-test comparing each promoter or enhancer sample to scrambled control (*** $p < 0.001$). (**B**–**D**) FACS sorting results of wildtype MDA MB-436 cells (**B**) compared to enhancer knockout cells (**C** and **D**) stained with propidium iodide and Annexin V-Alexa Fluor. The dot plots quantify the ratios of live (quadrant 4, Q4) vs apoptotic (quadrant 3, Q3) vs. dead (quadrant 2, Q2) cells.

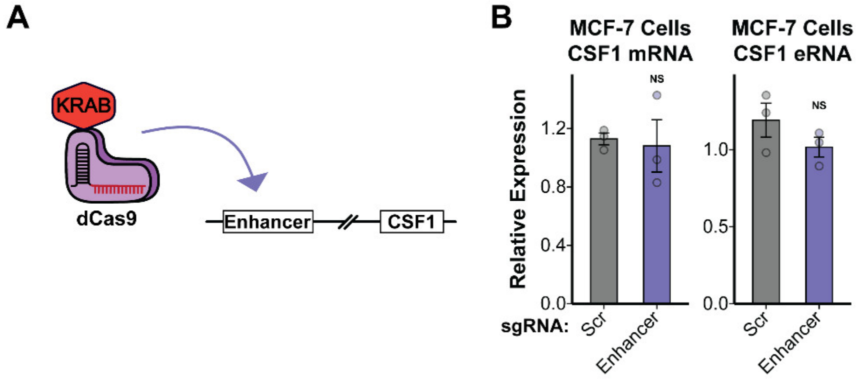

Figure S2. CSF1 mRNA expression after dCas9-KRAB perturbation of the enhancer in MCF-7 cells (related to Figure 3). (**A**) Diagram showing the targeting of dCas9-KRAB to the enhancer (purple) of CSF1. (**B**) qRT-PCR displaying the relative fold change in CSF1 mRNA and eRNA expression when targeting the CSF1 enhancer with dCas9-KRAB in MCF7 cells. Each bar represents the mean fold change (relative to a scrambled guide RNA) and each point shows the individual fold change per replicate. Error bars show standard error of the mean. Significance determined by a two-sided t-test comparing each promoter or enhancer sample to scrambled control (The designation of NS represents statistically not significant).

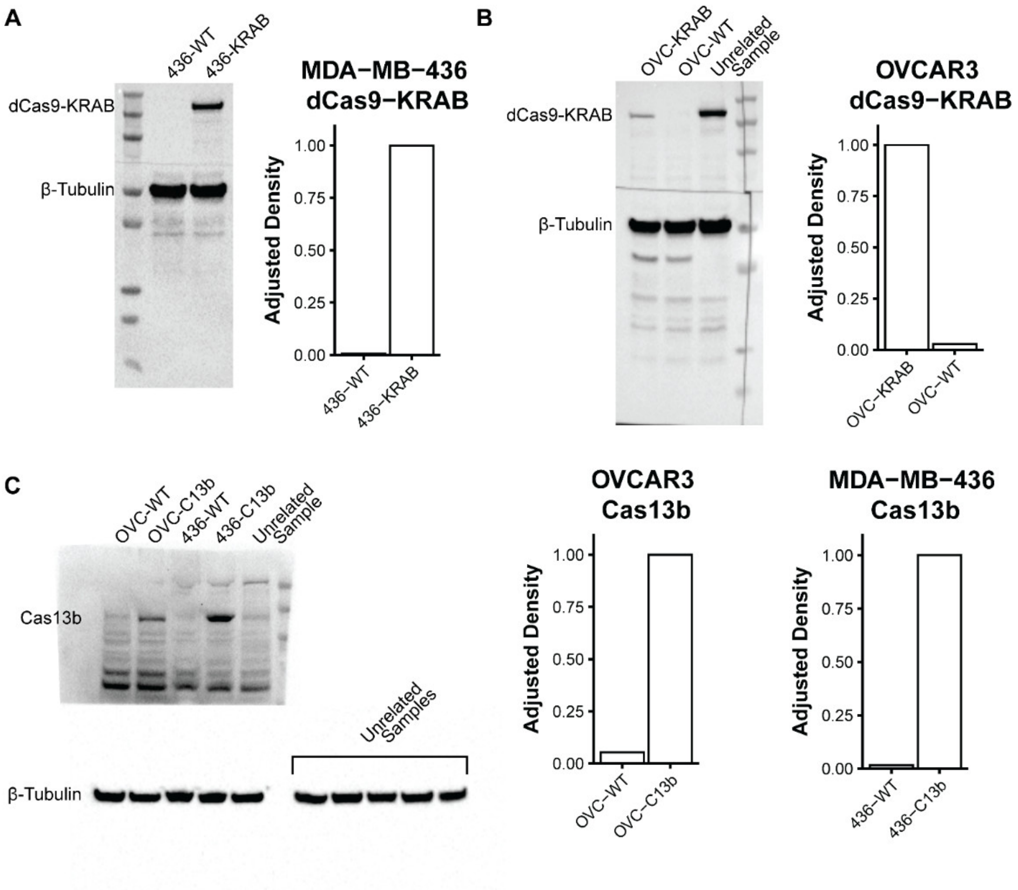

Figure S3. Original uncropped Western blots shown in main text (related to Figures 3–5). (**A**) Uncropped Western blot and densitometry analysis associated with Figure 3B. (**B**) Uncropped Western blot and densitometry analysis associated with Figure 5C. (**C**) Uncropped Western blot and densitometry analysis associated with Figure 4B (MDA-MB-436 Cas13b) and Figure 5D (OVCAR3 Cas13b).
